# Assessing Parent Behaviours in Parent–Child Interactions with Deaf and Hard of Hearing Infants Aged 0–3 Years: A Systematic Review

**DOI:** 10.3390/jcm10153345

**Published:** 2021-07-29

**Authors:** Martina Curtin, Evelien Dirks, Madeline Cruice, Rosalind Herman, Lauren Newman, Lucy Rodgers, Gary Morgan

**Affiliations:** 1 Speech and Language Therapy (Paediatrics, Community), Homerton University Hospital NHS Foundation Trust, London E9 6SR, UK; 2 Language and Communication Science Division, University of London, London EC1 0HB, UK; m.cruice@city.ac.uk (M.C.); r.c.herman@city.ac.uk (R.H.); g.morgan@city.ac.uk (G.M.); 3 Dutch Foundation for the Deaf and Hard of Hearing Child, 1073 GX Amsterdam, The Netherlands; edirks@nsdsk.nl; 4 Department of Psychology, Utrecht University, 3584 CH Utrecht, The Netherlands; 5 Barts Health NHS Trust, London E1 2ES, UK; lauren.newman3@nhs.net; 6 Sussex Community NHS Foundation Trust, Brighton BN2 3EW, UK; lucy.rodgers@ucl.ac.uk

**Keywords:** deaf, parent–child interaction, assessment, early interaction, speech and language therapy, clinical research

## Abstract

Background: Despite early identification and advancements in cochlear implant and hearing aid technology, delays in language skills in deaf children continue to exist. Good-quality parent–child interaction (PCI) is a key predictor for the successful development of deaf children’s signed and/or spoken language. Though professionals have standard assessments to monitor child language, a clinical tool to observe the quality of parental interaction is yet to be developed. Aims and methods: This systematic review with narrative synthesis aims to uncover which parent behaviours are assessed in PCI studies with deaf infants aged 0–3 years, how these behaviours are assessed, and which are correlated with higher scores in child language. Results: Sixty-one papers were included, spanning 40 years of research. Research included in the review assessed parents’ skills in gaining attention, joint engagement, emotional sensitivity, and language input. PCI was mostly assessed using coding systems and frame-by-frame video analysis. Some of the parent behaviours mentioned previously are associated with more words produced by deaf children. Conclusion: The results of the review provide the evidence base required to develop the content of a future clinical assessment tool for parent–child interaction in deafness.

## 1. Introduction

Much research describes the importance of good-quality parent–child interaction for children’s language development [1]. Parents are seen as the main provider of the social and linguistic stimulation required for successful child language development [2].

Children develop the foundations of language through the ‘serve and return’ of communicative interactions with their caregiver. This happens first through vocal and visual means (exclamations, babbling eye contact, facial expressions, gestures and pointing) and then through language use [3]. Parents scaffold this development through prompts and contingent reactions to their child’s behaviours [4]. These behaviours in turn encourage and reinforce a child’s communicative intentions [5]. For example, relationships have been found between a parent’s responses to child gesture and vocalisation, and child vocabulary development [6].

### 1.1. Parent–Child Interaction (PCI) and Deafness

Deafness impacts the child’s ability to access spoken language. However, with 95% of deaf children born to hearing families, this can bring challenges for successful communication, and long-term consequences for language development and academic success [7]. In this paper, we use the term ‘deaf’ to refer to all levels of deafness, from mild to profound. We also follow the recommendation from the British Association of Teachers of the Deaf and use the terms ‘deafness’ and ‘deaf’ rather than ‘hearing loss’ and ‘hearing impairment’ [8]. In addition, this review is intended to be inclusive of deaf children developing signed and/or spoken language.

Despite earlier identification and advancements in hearing aid and cochlear implant technology, delays in receptive and expressive language skills in deaf children continue to exist [9,10]. Many studies have found the quantity and quality of parental interaction to be one of the main predictors of deaf children’s language outcomes [11,12,13,14,15]. Parents who have not yet developed skills in effectively communicating with their deaf child may provide lower-quality language input [16], which in turn affects the child’s language development.

To illustrate, studies have reported that hearing parents of deaf children can often be more directive in their interactions compared to deaf parents of deaf children and hearing parents of hearing children [9,17]. This manifests as increased interruptions to the child’s attention by parents initiating new, unrelated activities [18]. Hearing parents also elicit language from their deaf child through requests rather than conversations, meaning deaf children have less experience of two-way interaction and receive less feedback on their communicative attempts [19]. An important foundation for language development is joint attention, i.e., two people with a mutual focus. Hearing parents struggle to establish and maintain this behaviour with their young deaf infants [20,21]. Higher rates of directive behaviours from hearing parents of deaf children [17,22], are less conducive for maintaining attention. The mismatch of hearing status means that hearing parents need to adapt their communication skills to attain successful joint engagement in by gaining or waiting for the deaf child’s attention before starting to communicate and sequentially shifting attention between the environment/objects and each other. In comparison, deaf parents are using these social engagement strategies at an early age with their deaf infants [23,24] and we see an earlier tuning in of the deaf child’s gaze [25].

### 1.2. Improving Parent’s Skills in Interaction

Despite the association between parental interaction and child language development, enhancing hearing parents’ communication with deaf children is a complex issue. Parents of deaf children receive multiple home visits and attend appointments for medical and audiological purposes where they learn about deafness, communication, and future education [26]. To reduce the communication difficulties that can cause increased family stress [27], hearing parents are required to adapt their communication style and often receive family-centred interventions that incorporate new strategies to enhance their interaction skills. The level of parental involvement in these interventions varies and can be associated with acceptance of the child’s deafness, parental self-efficacy, and the amount of support a family receives [28].

In wider research, the impact of parent-implemented interventions on at-risk children within hearing populations is well documented in Autism Spectrum Disorders [29], Developmental Language Disorder [30], stuttering [31] and in families with low socio-economic status [32]. There has been less research on the effect of PCI interventions in deafness, with some studies suggesting that parents who received intervention had deaf children with better communication and/or language skills [13,33,34].

### 1.3. The Assessment of Parent–Child Interaction (PCI)

As PCI is important, it is necessary to have valid and reliable tools to assess it. Each parent and child is different, and clinical time spent assessing each individual’s characteristics is important for target setting, as well as tailoring and implementing the intervention successfully.

Research studies of PCI typically use video recordings and analyse pre-determined target behaviours, i.e., the behaviours of the parent and child, and the reciprocity between them. However, there is much variability across studies in the analysis of this interaction and the use of the same tools across studies is sparse. For example, a recent systematic review identified more than 500 observational tools used for measuring PCI [35]. The population of focus in Lotzin et al. was parents with infants aged 0 to 12 months and was not specific to deafness. Lotzin et al. concluded that only 24 of these tools met their criteria of being psychometrically tested and published in peer-reviewed journals. The authors further highlighted that only 10 tools provided evidence in 4 out of 5 domains of validity; tools often lacked a user manual, were based on interactions from samples in North America and Western Europe only, and were not thoroughly validated on fathers [35]. The authors recommend that researchers and clinicians should use tools with some evidence of validity.

### 1.4. The Current Study

A paper by Moeller and colleagues entitled ‘Best Practices in Family-Centered Early Intervention for Children Who Are Deaf or Hard of Hearing’ [36] highlighted the importance of parents’ interactions and the need for regular assessment of PCI. Yet, to our knowledge, there is no valid clinical assessment tool that evaluates a parent’s interaction skills when they are communicating with their deaf child.

The current systematic review forms part of a larger project to develop an assessment tool for PCI in deafness and aims to address the following three research questions. It is the first review to synthesise all the available evidence on the following three questions:

(1) Which parent behaviours are being assessed in parent–child interaction studies in deafness for infants aged 0–3 years?
(2) How are parent behaviours being assessed?
(3) Which parent behaviours are associated with higher child language scores?

## 2. Materials and Methods

This systematic review was conducted following guidance from the Cochrane Handbook for Systematic Reviews. The Preferred Reporting Items for Systematic Reviews and Meta-Analyses (PRISMA) Statement [37] was used to ensure robust reporting. The review protocol was uploaded to PROSPERO, ref: CRD42020198567. The research team and expert advisory board agreed on and approved the protocol.

### 2.1. Selection Criteria

For this work, all peer-reviewed, published studies available in English that included deaf infants aged 0–3 years with any level of deafness, any amplification (or none) and any communication modality were included. Parents could be hearing or deaf. Included papers had to investigate free, unstructured play between the deaf child and parent in any context (i.e., in a home, lab or clinic). Play was selected as this is often the activity observed in professional practice. Parent–child dyads had to be video recorded, with video data used for the analysis.

All study types (quantitative, qualitative or mixed methods) and any research designs (RCT, intervention or observational studies) were included. PCI assessment had to be objectively measured by the study research team, through non-validated and/or validated measures. Results of papers had to report on parent behaviours and interaction.

Papers were excluded if: they used subjective data (i.e., parent self-report) to analyse PCI; only reported on child behaviours; included children who were deaf and also had either Autism Spectrum Disorder or a Visual Impairment (as parent behaviours and strategies may be significantly different within this sub-population); or only analysed verbal recordings. The latter is because PCI, particularly in deafness, is more than spoken words a parent says. It also encompasses how parents engage with their child though eye contact, facial expression, touch and gesture. These aspects of PCI are all important parts of the language learning process. As such, this last criterion ensured research studies that assessed these behaviours as well as language-based communication (spoken and signed), were captured in the review.

The first author and an information specialist librarian searched the following eight databases on 26 June 2020: Medline, PsycINFO, CINAHL, Communication Source, Cochrane Databases, Embase, Web of Science and Scopus, through two platforms: Ovid and EBSCOhost.

### 2.2. Search Strategy

Synonyms were used for ‘deaf’, ‘infant’, ‘parent’ and ‘interaction’. Please see Appendix A for full search strategy.

### 2.3. Selection Process

Covidence software was used in the review and data collection process. All search results were uploaded, and duplicates were automatically removed. As an initial trial, 30 papers (1% of the search results) were reviewed independently by authors M.C. (Martina Curtin) and E.D., with arising conflicts discussed. Each paper was then independently reviewed for inclusion based on article title and abstract, with authors M.C. (Madeline Cruice) and E.D. achieving 95% agreement (k = 0.64) at this stage.

Full texts were retrieved for the articles that met the inclusion criteria. Despite contacting authors, 5% were unable to be retrieved. Each paper was independently reviewed by M.C. (Martina Curtin) and E.D. Discrepancies were resolved every 1 to 2 weeks. Authors met 82% agreement (k = 0.59).

### 2.4. Data Collection Process

Each paper included in the review was independently extracted and reviewed by the first author and at least one other author. All authors were involved in meetings to gain consensus, check discrepancies, and make final decisions.

### 2.5. Data Items

The extraction form (Appendix B) was written by the first author, then reviewed and amended by the other authors and members of the project advisory board, before being added to Covidence.

To answer research questions 1 and 2, the main outcome variables included information about the PCI assessment including which behaviours were assessed and how the interaction was assessed (i.e., where, for how long and how often) and by what means (i.e., coding systems or scales, by whom and what reliability information). The results of the assessments were also collected to answer research question 3.

In order to provide an overview of the papers included in the review, the following variables were included on the extraction form: study characteristics (country of study, research design, aims, conflicts declared) and participant characteristics (child age, deafness level, amplification used, communication mode used, parent age, social economic status, parent education level). This detailed information gives us an understanding of the similarities and differences between studies, their applicability to populations seen clinically, as well as highlighting repeated samples.

Information on intervention characteristics (intervention name, delivery, dose) were also extracted. Finally, conclusions, confounding factors, and limitations identified by the authors were also extracted. Whilst not reported in our review, these were used to verify the results of the analysis and assist our assessments of bias. Missing information was labelled as ‘not reported’.

### 2.6. Risk of Bias Assessment

The Joanna Briggs Institute Critical Appraisal Checklist for Cross-Sectional Studies [38] was used as the Risk of Bias Assessment for all the observational studies. For intervention studies, the ROBINS-I [39] was used. No adaptations were made to either tool. Both tools included an overall risk of bias judgement at the end with guidance on how to reach this. Similar to data extraction, each study’s risk of bias was independently reviewed by the first author and at least one other author, with differences resolved in regular meetings.

### 2.7. Synthesis (Preparation and Approach)

Extracted data were exported into Excel from Covidence and a table summarising the included papers was created. Table 1 outlines the key features of each paper in relation to the review’s questions (parent behaviours assessed, methods of assessment and whether child language was assessed). We also indicate the risk of bias outcome for each study.

Included papers were then grouped into sub-sets of conceptually similar PCI behaviours. Due to the qualitative nature of the extracted data, a narrative synthesis approach [40] was taken using extra guidance on concept mapping [41].

**Table 1:** Papers included in the review (*n* = 61) and the associated research questions.

Paper No	First Author	Year	Reported Country of Study	Study Design	Degree of Hearing Loss	No of Dyads	PCI Behaviours Assessed	PCI Measure (Method)	Child Lang Assessed?	Risk of Bias
1	Beatrijs. W., et al. [23]	2019	Belgium	Two between-groups, observational studies	No Report	13	Attention-Getting Strategies	Coding	N	Moderate
2	DesJardin, J. L. [42]	2006	USA	Within-group, observational study	Mod–Prof	32	Attention-Getting Strategies and Parental Communication	Coding	Y	Moderate
3	Loots, G. et al. [43]	2003	Belgium	Between-groups, observational study	Mod–Prof	33	Attention-Getting Strategies	Coding	N	Low
4	Waxman, R. et al. [44]	1997	USA	Between-groups, observational study	Mild–Prof	77	Attention-Getting Strategies	Coding	N	Moderate
5	Chasin, J. et al. [45]	2008	UK	Between-groups, observational study	Profound	9	Attention-Getting Strategies and Child Eye Gaze	Coding	N	Moderate
6	Harris, M. et al. [46]	1989	UK	Within-group, longitudinal observational case series	Profound	4	Attention-Getting Strategies and Child Eye Gaze	Coding	Y	Serious
7	Harris, M. et al. [47]	1997	Australia and UK	Between-groups, observational study	Profound	11	Attention-Getting Strategies and Child Eye Gaze	Coding	N	Critical
8	Harris, M. et al. [47]	2005	UK	Between-groups, observational study	Profound	26	Attention-Getting Strategies and Child Eye Gaze	Coding	N	Moderate
9	Lederberg, A. R. et al. [48]	1998	USA	Between-groups, observational study	Sev–Prof	40	Attention-Getting Strategies and Child Eye Gaze	Coding	Y	Moderate
10	Prendergast, S. G. et al. [49]	1996	USA	Between-groups, observational study	Sev–Prof	16	Attention-Getting Strategies and Child Eye Gaze	Coding	N	Moderate
11	Gabouer, A. et al. [50]	2018	USA	Between-groups, observational study	Sev–Prof	18	Attention-Getting Strategies and Joint Engagement	Coding	N	Serious
12	Loots, G. et al. [24]	2005	Belgium	Between-groups, observational study	Mod–Prof	31	Attention-Getting Strategies and Joint Engagement	Coding	N	Low
13	Nowakowski, M. et al. [51]	2009	Canada	Between-groups, observational study	Sev–Prof	56	Attention-Getting Strategies and Joint Engagement	Coding	Y	Moderate
14	Tasker, S. et al. [52]	2010	Canada	Between-groups, observational study	Sev–Prof	53	Attention-Getting Strategies and Joint Engagement	Coding	Y	Low
15	Barker, D. H et al. [9]	2009	USA	Between-groups, observational study	Sev–Prof	185	Joint Engagement	Coding	Y	Low
16	Cejas, I. et al. [10]	2014	USA	Between-groups, observational study	Sev–Prof	276	Joint Engagement	Coding	Y	Moderate
17	Roos, C. et al. [53]	2016	Sweden	Within-group, observational study	Sev–Prof	12	Joint Engagement	Coding	N	Moderate
18	Spencer, P. E. [54]	2000	USA	Between-groups, observational study	Mod–Prof	80	Joint Engagement	Coding	N	Serious
19	Dirks, E. et al. [20]	2019	The Netherlands	Between-groups, observational study	Mod	51	Joint Engagement and Parental Sensitivity	Existing Scale + Coding	Y	Low
20	Gale, E. et al. [55]	2009	USA	Between-groups, observational study	Sev–Prof	15	Joint Engagement and Parental Sensitivity	Coding	Y	Moderate
21	Janjua, F. et al. [56]	2002	UK	Within-group, observational study	Sev–Prof	13	Joint Engagement and Parental Sensitivity	Coding	Y	Serious
22	Lederberg, A. R. et al. [57]	1990	USA	Between-groups, observational study	Mild–Prof	82	Joint Engagement and Parental Sensitivity	Novel Scale + Coding	Y	Moderate
23	Meadow-Orlans, K. P. et al. [58]	1993	USA	Between-groups, observational study	Mod–Prof	80	Joint Engagement and Parental Sensitivity	Novel Scale + Coding	N	Moderate
24	Meadow-Orlans, K. P. et al. [18]	1996	USA	Between-groups, observational study	Mod–Prof	80	Joint Engagement and Parental Sensitivity	Novel Scale + Coding	N	Moderate
25	Abu Bakar, Z. et al. [59]	2010	Not reported	Between-groups, observational study	Sev–Prof	18	Parental Sensitivity	Novel Scale	N	Serious
26	Meadow-Orlans, K. P. et al. [60]	1995	USA	Within-group, observational study	Mild–Prof	43	Parental Sensitivity	Novel Scales	N	Moderate
27	Lam-Cassettari, C. et al. [61]	2015	UK	Between-groups, intervention study	Mod–Prof	14	Parental Sensitivity	Existing Scale	N	Moderate
28	Meadow-Orlans, K. P. [62]	1997	USA	Between-groups, observational study	Mod–Prof	40	Parental Sensitivity	Novel Scales	N	Moderate
29	Pressman, L. J. et al. [63]	1998	USA	Between-groups, observational study	Mild–Prof	42	Parental Sensitivity	Existing Scale	Y	Moderate
30	Pressman, L. J. et al. [64]	1999	USA	Between-groups, observational study	Mild–Prof	24	Parental Sensitivity	Existing Scale	Y	Low
31	Spencer, P.E. [65]	1996	USA	Between-groups, observational study	Mod–Prof	43	Parental Sensitivity	Novel Scale	Y	Low
32	Vohr, B. et al. [66]	2010	USA	Between-groups, observational study	Mild–Prof	58	Parental Sensitivity	Existing Scale	Y	Low
33	Waxman, R. et al. [67]	1996	USA	Between-groups, observational study	Mod–Prof	30	Parental Sensitivity	Coding	N	Moderate
34	Ambrose, S. E. [68]	2016	USA	Between-groups, observational study	Mild–Prof	48	Parental Sensitivity	Coding	Y	Low
35	Caissie, R. et al. [69]	1993	Not reported.	Between-groups, observational study	Sev–Prof	11	Parental Sensitivity	Coding	Y	Serious
36	Eddy, J. R. [70]	1997	Australia	Between-groups, observational study	Sev–Prof	18	Parental Sensitivity	Coding	Y	Serious
37	Glanemann, R. et al. [33]	2013	Germany	Between-groups, intervention study	Mod–Prof	29	Parental Sensitivity	Coding	Y	Moderate
38	Wedell-Monnig, J.; et al. [71]	1980	USA	Between-groups, observational study	Sev–Prof	12	Parental Sensitivity	Coding	N	Serious
39	MacTurk, R. H. et al. [72]	1993	USA	Between-groups, observational study	Mod–Prof	40	Parental Sensitivity and Child Eye Gaze	Novel Scales	N	Serious
40	Choo, D. et al. [73]	2016	Australia	Within-group, observational study	Sev–Prof	12	Parental Sensitivity and Parental Communication (Comm.)	Novel Scale	N	Moderate
41	James, D. et al. [74]	2013	UK	Within-group, intervention study	Profound	3	Parental Sensitivity and Parental Comm.	Existing Scale + Coding	Y	Serious
42	Nicastri, M. et al. [13]	2020	Italy	Between-groups, intervention study	Profound	Not reported: 22 parents of 14 children	Parental Sensitivity and Parental Comm.	Existing Scale	Y	Moderate
43	Preisler, G. M. [75]	1995	Sweden	Within-group, observational study	No Report	14	Parental Sensitivity and Parental Comm.	Coding	N	Serious
44	Quittner, A. L. et al. [14]	2013	USA	Between-groups, intervention study	Sev–Prof	285	Parental Sensitivity and Parental Comm.	Scales (×2 existing, ×1 novel)	Y	Low
45	Quittner, A. L. et al. [76]	2016	USA	Between-groups, observational study	Profound	285	Parental Sensitivity and Parental Comm.	Scales (×1 existing/×1 novel)	Y	Low
46	Ahmad, A. et al. [77]	2016	Australia	Between-groups, observational study	Mild–Prof	16	Parental Communication	Coding	N	Moderate
47	Brown, P. M. et al. [78]	2004	Australia	Between-groups, observational study	Profound	20	Play and Parental Communication	Coding	Y	Moderate
48	Chen, D. [79]	1996	USA	Between-groups, observational study	Mod–Prof	12	Parental Communication	Coding	Y	Serious
49	DeVilliers, J. et al. [80]	1993	USA	Within-group, observational study	Profound	2	Parental Communication	Coding	N	Critical
50	Morelock, M. et al. [81]	2003	USA/Australia	Between-groups, observational study	Profound	9	Parental Communication	Coding	N	Serious
51	Roberts, M. [34]	2019	USA	Randomised controlled trial	Mod–Prof	19	Parental Communication	Coding	Y	Moderate
52	Koester, L. S. et al. [82]	2010	USA	Between-groups, observational study	Mod–Prof	61	Parental Communication	Coding	N	Serious
53	Paradis, G. et al. [83]	2015	USA	Between-groups, observational study	No Report	60	Touch and Parental Sensitivity	Existing Scale + Coding	N	Moderate
54	Pipp-Siegel, S. et al. [84]	1998	USA	Between-groups, observational study	Mild–Prof	48	Touch and Parental Sensitivity	Existing Scale + Coding	N	Moderate
55	Abu-Zhaya, R. et al. [85]	2019	USA.	Between-groups, observational study	Mild–Prof	24	Touch	Coding	N	Moderate
56	Gabouer, A. et al. [86]	2020	USA	Between-groups, intervention study	Sev–Prof	18	Touch	Coding	N	Serious
57	Spencer, P.E. [87]	1993a	USA	Between-groups, observational study	Mod–Prof	36	Other: Maternal Comm. Modality	Coding	Y	Low
58	Spencer, P.E. [88]	1993b	USA	Between-groups, observational study	Mod–Prof	7	Other: Maternal Comm. Modality	Coding	Y	Moderate
59	Lederberg, A. R. et al. [89]	2000	USA	Between-groups, observational study	Sev–Prof	40	Other: Maternal Comm. Modality	Coding	Y	Moderate
60	Depowski, N. et al. [90]	2015	USA	Between-groups, observational study	Sev–Prof	8	Other: Type and Use of Gesture	Coding	N	Serious
61	Lieberman, A. et al. [91]	2014	USA	Between-groups, observational study	Mod–Prof	8	Other: Maternal and Child Eye Gaze	Coding	Y	Moderate

## 3. Results

### 3.1. Study Characteristics

**Figure 1:**
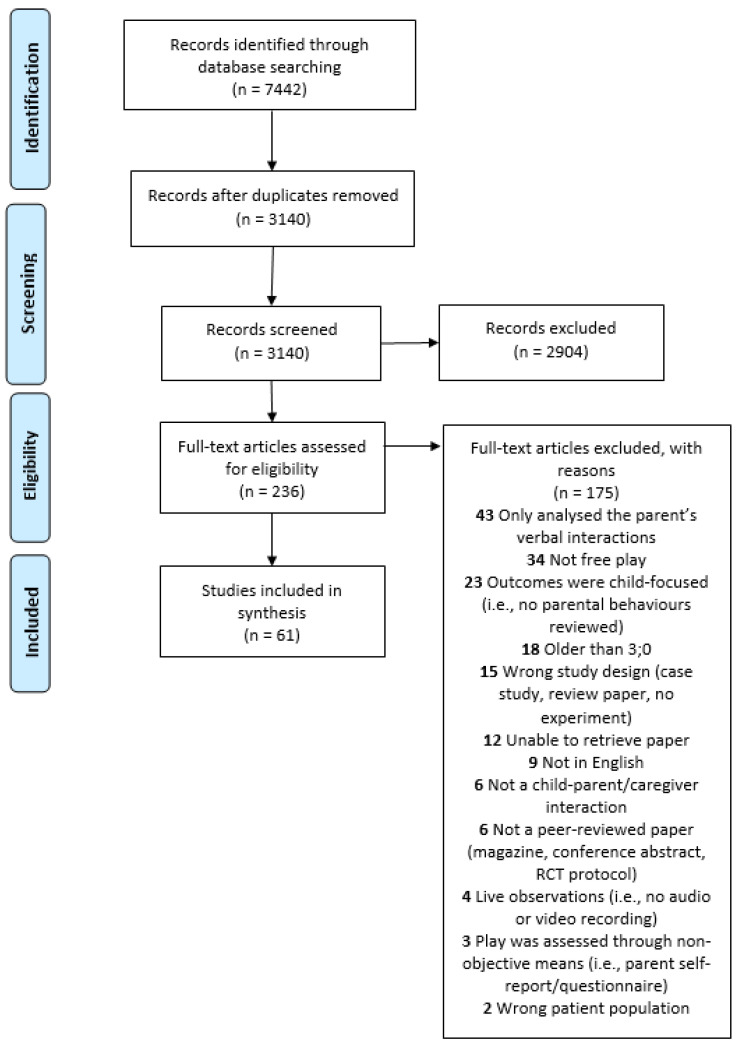
PRISMA systematic review flow diagram.

In total, 3140 papers were identified and included in the selection process. Following title and abstract screening, 226 papers were retrieved for the full-text review. After in-depth reading, 61 papers were included in this review. See PRISMA [37] flowchart (Figure 1) for more details.

Included papers spanned 40 years of research (1980–2020). Most studies were from the USA (63%), followed by Europe (21%), Australia (10%), Canada (3%) or unreported (3%). The most common research design was between-groups, observational studies (75%), followed by within-group, observational studies (11%), between-groups intervention studies (8%) and one randomised control trial (2%), one case series (2%) and one within-group intervention study (2%).

Ten papers (16%) observed hearing parents of deaf children exclusively and three papers (5%) observed deaf parents of deaf children exclusively. The remaining 48 papers (79%) recruited two or more of the following groups for comparisons: hearing parents of deaf children (compared alongside other groups in 46 of 61 papers), deaf parents of deaf children (compared alongside other groups in 19 of 61), deaf parents of hearing children (compared alongside other groups in 7 of 61) and hearing parents of hearing children (compared alongside other groups in 40 of 61).

Many papers did not report key demographic information relating to the children in the studies: child communication mode (signing, speaking, etc.) was not reported in 28% of papers; presence of additional needs was not reported in 27% of papers; ethnicity was not reported in 53% and social economic status was not reported in 73% of papers. Insufficient descriptions of the samples were thus considered as potential bias. When this demographic information was reported, included studies indicated that children used a mix of speech and sign in their productive language (33% of papers), were either typically developing or no known additional needs were present (56%), and were Caucasian (42%). Research studies were mainly focussed on mother-child interaction (75% of papers); a small number of papers also included fathers (8%) and the remaining papers did not explicitly state which parent was involved.

### 3.2. Quality Assessment

Seventy percent of the papers in this review achieved a low (*n* = 12) or moderate (*n* = 31) level of bias rating. Thirty percent of papers were rated as having a serious (*n* = 16) or critical risk of bias (*n* = 2). Papers were generally evaluated with higher levels of bias for not providing enough detail in the description of participants, the assessment procedures, reliability checks, and not using statistical tests for comparisons. Whilst we removed these papers from our analysis to answer research question 3 (correlations between PCI and language outcomes), we felt that it was appropriate to leave these papers in the analysis for the methodology-based research questions 1 and 2 and present each paper’s risk of bias in Table 1.

Sample size differed across the papers but was low in comparison to PCI studies with hearing populations. The average sample size of parent–child dyads was 45, with a range of 2–285 and a mode of 18. Sarant and colleagues state: “Large numbers of participants must be included in order to draw valid conclusions. However, this is difficult to accomplish in the case of children with hearing loss because hearing loss is a relatively rare condition and many research centers do not have the resources to conduct large population studies [92] (p. 206).”

### 3.3. Research Question 1: Which Parent Behaviours Are Being Assessed in PCI Studies in Deaf Infants Aged 0–3 Years?

We found that research studies assessed parents on gaining their deaf child’s attention, maintaining joint engagement, levels of parental sensitivity, and parental communication behaviours. Each of these will now be explored in greater detail.

#### 3.3.1. Attention-Getting Behaviours

Attention-getting behaviours can be defined as explicit bids, made by the parent, with the intent of gaining or directing their deaf child’s attention. The bid for attention can use one or more modalities. Fourteen (14) of the 61 studies (papers 1 to 14 in Table 1) observed this aspect of parent behaviour. Data from these papers have been synthesised into four modalities: visual, auditory, tactile, and multi-modal.

##### Visual Strategies

Using any of the following within the child’s visual field with the intention to gain or direct the child’s attention: waving, gesturing, reaching, pointing, making eye contact, switching gaze between an object and the child, holding or moving an object or toy directly into the child’s visual field, offering an object, manipulating an object, demonstrating play with toys, making faces, displacing the location of a sign into the child’s vision or signing space, and changing affect.

##### Auditory Strategies

Using any of the following sounds to gain a child’s attention: using voice to call the child’s name, using a word such as ‘look!’ or non-words (e.g., ‘whee’ or ‘pssst!’), humming or singing; use of the body to make sounds (outside of the child’s visual field) such as clapping or clicking; and/or the use of toys or objects to make sounds.

##### Tactile Strategies

Using any of the following to gain or direct a child’s attention: making gestures or signs on the body of the child; tapping, touching, hugging, or holding the child; grabbing on to the child’s clothing; moving the child’s limbs; and touching the child with a toy (out of their visual field). This category also includes tapping the ground to create vibrations, and physically adjusting the child’s position to direct their attention.

##### Multi-Modal Cues

Combinations of the above—multi-modal cues—were also coded. For auditory-visual combinations, a parent might say ‘uh oh!’ and gesture as a toy rolls under the table. For visual-tactile, a parent may turn a child sat on their lap and then point to a new toy out of their current visual field. Other combinations may be auditory-visual-tactile, e.g., holding a child while talking to them and pointing to a toy.

Coding in papers that included deaf parents of deaf children also featured ‘waiting’ as an attention-getting strategy [1,3,12,17], e.g., it was noted when parents did not initiate the interaction or any expression but actively waited until their child was looking at them before communicating. This could be seen as an attention-getting strategy, as a paused action may warrant the child to look towards the parent. These papers also put greater focus on parents’ visual-tactile attention-getting strategies (ibid).

Related to interaction, six studies (papers 5–10 in Table 1) reported on the success of parental attention-getting behaviours in relation to child gaze and noted gaze could be either elicited, responsive, spontaneous, and failed.

Papers 11 to 14 in Table 1 combined attention-getting behaviours with joint engagement between parent and child. This phenomenon was the focus for many more papers included in this review and is defined and described in the next section.

#### 3.3.2. Joint Engagement

Joint engagement is a state of mutual focus and shared involvement between a parent and child, where both participate in reciprocal, contingent, socially directed behaviours. Authors use the following terms interchangeably: joint engagement, joint attention, and intersubjectivity, with frequent references to the coding systems of Bakeman and Adamson [93], Prezbindowski and colleagues [94], and Tasker and Schmidt [95]. Twelve of the studies observed this phenomenon (papers 11 to 24 in Table 1).

##### When Engagement Is Established and When It Is Terminated

Marking joint engagement as ‘established’ varied from three seconds of mutual focus to a five-second rule of engagement (where the child had to respond to a parent’s act within five seconds). It was also categorised as three or four sequential, on-topic, connected turns where both the parent and child’s attention and/or language are focussed on the same event or object. Physical acts were also included (such as tickling or laughing). Similarly, how to class a state of joint engagement as finished also varied across papers. Joint engagement was ‘terminated’ when one social partner stopped responding and their attention was lost after a set time period which varied between papers from 3 to 15 s.

##### Levels of Joint Engagement

Some authors differentiated between ‘supported/passive’ joint engagement, with the parent joining the child in an activity and helping to support the joint engagement, without the child acknowledging the parent, and ‘coordinated’, wherein both parent and child exclusively engaged with each other and the activity. Interactions may be physical and/or visual (body movements, facial expressions, tickles) or may be ‘symbol-infused’, which refers to the use of language (signed, spoken or referential gesture) within a period of joint engagement.

The authors of the current review use ‘joint engagement’ as the term suggests parent and child are active participants, doing more than simply attending to the same thing.

#### 3.3.3. Parental Sensitivity

Parental sensitivity refers to a set of skills that enables a parent to be emotionally connected, in tune and responsive to their child’s needs, goals, and communicative attempts. A parent with a high level of sensitivity will be positive and accepting of their child and will strive for interactive congruence. Twenty-five (25) studies within the review assessed this aspect of parent behaviour (papers 19 to 45 in Table 1) and therefore it is the most frequently assessed aspect of PCI. Across papers parental sensitivity was described as a group of sub-skills. Parents were often assessed on each of these sub-skills using Likert-scales. These behaviours consisted:

##### Positive Regard

A parent showing enthusiasm, warmth, pleasure, love, and respect for their child, regularly using positive body language, praise and comforting and playful physical touch. Opposite: Covert or overt hostility, negative affect, physical harshness.

##### Availability

A parent who is genuinely interested and actively involved in participating in accessible interactions with their child. Opposite: Passive, bored, and disengaged.

##### Contingent and Responsive

A parent that follows their child’s lead and pace and responds with contingent, on-topic behaviours or language. Opposite: Directive, intrusive, dominant, and regularly initiating new topics.

##### Emotionally Sensitive

A parent who is emotionally attuned and adaptive. Able to recognise and respond to distress and disinterest, and repair or resolve misunderstandings or conflict. Opposite: Lacking or unhelpful emotional responses, unwillingness to soothe or resolve incidents causing discomfort.

##### Structure and Stimulation

A parent who is able to support a child’s interest by guiding and developing the interaction with appropriate play and language. The parent will be flexible and accept a change in play or routine put forward by the child. Opposite: Overpowering, structuring the play, inappropriate pace or activity, highly authoritative, inflexible, or formally teaching the child.

##### Consistency

A parent who can absorb a range of child emotions and behaviours, whilst remaining mostly constant in their behaviours, predominantly striving for a positive interaction. Opposite: Unpredictable behaviour that changes regularly in the interactions from positive to negative.

Most of the papers also included a rating of the child’s levels of responsiveness (also termed compliance or eagerness to respond) and involvement (initiations made, willingness to share). Some codes and scales rated the dyads for overall synchronicity, reciprocity, enjoyment, and communicative competence (understanding of one another). Papers 39 to 44 assessed parental sensitivity along with parental communication behaviours.

#### 3.3.4. Parental Communication Behaviours

Parental communication behaviours are language-focused strategies used by parents during moments of interaction with their deaf child. Though most are explicitly linked to exposing the child to signed or spoken language, some behaviours are centred around increasing the child’s *access* to spoken or signed language. Papers that *only* recorded and analysed parent’s verbal interactions were excluded (*n* = 43) and are listed in Appendix C. Thirteen papers assessed parents on a range of communicative behaviours (papers 40–52 in Table 1) and these are summarised below:

**Increased access to language:** Some papers assessed parents on their ability to communicate within the child’s line of sight or whilst being face to face; others observed parents’ use of timing, i.e., waiting for the child to look before communicating. Others observed parents’ use of child-directed speech or child-directed sign, i.e., where parents modify their speech or sign to be more child orientated. For example, a parent may adapt the palm orientation of a sign so the child can see more of the hand; they may increase the size and range of movement of signs, they may exaggerate the non-manual features of accompanying signs (facial expressions), use exaggerated vocal pitch or acoustic/sign highlighting, where the parent adapts their amplification of words or signs closer to the child.

**Language input:** This category refers to signed or spoken languages. Similar to parental sensitivity, parents were assessed on their contingent talk and number of connected turns, as well as their off-topic initiations (i.e., directives, requests and questions). Parents’ use of language stimulation was coded and assessed on how they: labelled items or feelings; commented; described; made accompanying sounds; interpreted their child’s behaviour with language; repeated their child’s utterance; expanded their child’s language by adding 1 or 2 new words, or rephrased it with correct grammar. Parents’ mean length of utterance (MLU) was assessed in one paper. Parents’ use of praise, affirmation and encouragement was assessed through language use, intonation, their gesture, and facial expressions. Assessment of less frequent behaviours included the parent modelling play, and the parent opposing the child, either by rejecting their communication, correcting their communication, or prohibiting their child’s behaviour.

#### 3.3.5. Use of Touch

The frequency and function of parents’ use of touch when interacting with their child was also assessed in a small set of studies (Papers 53–56 in Table 1). The authors of these papers were interested in the type, location, and duration of parent-initiated touch. One paper looked at the temporal alignment between touch and parents’ utterances [85]. Two papers also measured parental sensitivity [83,84] with Paradis and Koester [83] creating a coding system to analyse the function of parental touch, e.g., affectionate, attention-getting and instructive.

#### 3.3.6. Other

Five papers included in the review sit within this category (papers 57–61 in Table 1). Three papers [87,88,89] focused on the communication mode of parents (i.e., how often they signed, used gesture or spoke). One paper looked in detail into the type and function of gesture used [90] and another transcribed American Sign Language and documented eye gaze between mother and child [91].

### 3.4. Research Question 2: How Are Parent Behaviours Assessed?

Most papers (66%, *n* = 40) used a coding system to assess PCI, often watching and coding films frame by frame, using software such as INTERACT (Mangold) and ELAN (Max Planck Institute). This method allowed an in-depth analysis of the behaviours focused on in RQ1. Thirteen (13) papers (22%) used Likert scales instead of coding and some scales were well-known and validated, while others were developed for the specific research study with little mention of pilot testing prior to their use. The Emotional Availability Scales [96] were used in 7 of the 11 papers that used validated scales (see Appendix D for a full list). Nine papers (12%) used a combination of coding and scales. See Table 2.

Fifty-one percent (31) of the papers used a one-off recording of PCI, whereas the remaining papers repeated filming as the child matured. Most videos were filmed in a lab (30%, *n* = 18), at home (26%, *n*= 16) or in a clinic (8%, *n* = 5). Some research studies used a mixture of the three settings (22%, *n* = 13). The remaining studies did not report the location of filming (6%, *n* = 4) or used a vague description (8%, *n* = 5), e.g., ‘a playroom’. The average length of film data made was 18.9 min, with recording length ranging from 3 to 60 min. Notably, 15% (9) of papers did not report on the length of the video made. In further detail, the average length of film used for analysis was 8.7 min, with a range of 1–20 min. Some papers specified that the whole recording was used for the scale data but only the central ten minutes was used for coding. In addition, other papers gave more general information related to how they selected the section of video for coding analysis, e.g., ‘five minutes into the recording’, ‘not the first few minutes’ or ‘the central five minutes’. However, some papers (26%, *n* = 16) did not report on the length of the video used for analysis.

Eighty (80) percent of papers (48/61) reported on reliability testing of the PCI assessment. On average, authors had 33% of their video tapes independently re-assessed by a second coder, with a range of 10–100% re-assessed. Thirty-nine papers (64%) used statistical reporting: 30% (18 papers) used Cohen’s kappa; 27% (16) used percentage of agreement; and 8% (5) used both calculations.

### 3.5. Research Question 3: Which Parent Behaviours Are Associated with Higher Child Language Scores?

In total, 46% (28) of papers assessed children’s language skills. To answer research question 3, the authors discounted nine papers (32% of 28 papers) that used informal measures of child language, such as coding of vocalisations and number of signs. We felt this increased the risk of bias and a valid measure could have been used. Therefore, 31% (19) of the 61 papers included at least one formal measure of child language skill. Four (21% of the 19 included) papers were removed due to serious or critical risk of bias due to a lack of demographic information and minimal reporting on reliability. One paper (5% of 19) was removed as the formal child language measure was used as a baseline characteristic rather than an outcome, and another paper (5% of 19) was removed due to the reporting of confounders in the results. From the remaining 13 papers (papers 2, 15, 16, 19, 20, 29, 30, 32, 34, 42, 44, 45 and 51 in Table 1) the MacArthur–Bates Communicative Development Inventory [97] was the most common formal language measure (used in 9 papers, 69% of 13), followed by the Reynell Developmental Language Scales [98] (used in 4 papers, 31% of 13). Across the papers, 1364 dyads were included initially but after removing repeated samples, the figure reduced to 803 dyads.

Before we explore the correlations between deaf children’s language and parent behaviours, it is worth noting that child hearing status or hearing level is one of the most significant predictors of language gain. Pressman and team [63] found that the hearing status of a child accounted for 34% of the variance in child language skill. The 13 papers (21% of 61) included in this section of the review covered a wide range of deafness; four papers (46% of 13) included mild to profoundly deaf children, five included severely to profoundly deaf children only (38% of 13), with other combinations and single levels of deafness (i.e., moderately deaf only) also included (*n*= 4, 15%).

Looking at the papers it is evident that capturing the impact of parent interaction on child language development is difficult within research with limited time. Seven of the 13 papers (54%) assessed PCI cross-sectionally at one time point (average child age 28.5 months). The remaining six longitudinal papers (46%) regularly assessed PCI with some studies following the child from 5 months through to 5 years of age.

From the 13 papers (21% of 61 papers) that included formal language assessments, the following parental behaviours were positively correlated with deaf children’s language.

#### 3.5.1. Joint Engagement

Higher child language scores were related to more time in higher level engagement states with a parent (i.e., coordinated joint engagement and symbol-infused joint engagement) [10]. Deaf children spent significantly less time in these states when compared to their hearing peers and therefore used less language [9,55].

Dirks and Rieffe [20] add further evidence to this finding: deaf children and their hearing parents are less successful in establishing joint engagement and have briefer episodes when they do. These authors found positive correlations between total duration of joint engagement and receptive and expressive language skills. Interestingly, Gale and Schick [55] and Dirks and Rieffe [20] found correlations between non-intrusiveness and joint engagement in mothers who followed their toddler’s interests, rather than directing, and this was also linked to more instances of joint engagement.

#### 3.5.2. Parental Sensitivity

Maternal sensitivity was positively correlated with expressive language and predicted language growth over time [14,76]. In their study of 285 deaf children with cochlear implants, Quittner and team found parents with above-average skills in maternal sensitivity and language stimulation had children with 1.52 years less of a language delay [14]. Dirks and Rieffe [20] also found positive relationships between parental sensitivity and receptive and expressive child language *and* total duration of joint engagement. Children with better language experienced longer interactions with their parents and this was linked to parents with higher levels of emotional sensitivity (ibid).

In their 1999 study, Pressman and colleagues found that maternal sensitivity was not correlated with children’s initial expressive language scores, but was positively correlated in their follow up assessments 12 months later [64]. In their regression analyses, maternal sensitivity positively predicted expressive language scores and accounted for 10% of the variance. In their 1998 study, they uncovered that maternal sensitivity had a larger positive effect on language in the sample of deaf children compared to their hearing sample [63].

Ambrose [68] focused on the responsiveness of mothers. She found that hearing mothers of deaf children were significantly less likely to respond to their child’s gestures, compared to mothers of hearing children. Despite having a similar number of gestures to their hearing peers, the deaf children used fewer words (ibid). This decrease in responsiveness may be associated with greater levels of maternal stress, as was found by Vohr et al. [66] where greater stress was related to decreases in positive regard, availability, enjoyment, and number of words produced by the child at 18–24 months.

#### 3.5.3. Parental Communication Behaviours

We remind the reader of our inclusion/exclusion criteria for this review: we included papers that explored correlations between parents’ verbal and visual behaviours and child language outcomes.

As such, for this section, we will not report on correlations found in DesJardin [42] (2006) or Quittner et al. [14,76], as parents’ communication was solely analysed in terms of their verbal input, despite other outcomes in their papers looking at features beyond spoken language (attention-getting behaviours and parental sensitivity, respectively). Therefore, only two papers [13,34] were included in this subsection. Both studies involved parents receiving training in PCI and both assessed deaf children’s pre-linguistic skills (i.e., pointing, co-ordinated joint engagement and gestures using the MacArthur Bates CDI Words and Gestures Form [97] or the Communication and Symbolic Behaviour Scale Behaviour Sample [99]

In the study from Nicastri et al. [13], parents received nine whole-group sessions and three individual sessions of training over 10.5 months. The intervention was based on the ‘It Takes Two to Talk’ Hanen program [100] and involved video modelling, where parents had opportunities to put their training into practice at home. Strategies within the program included waiting and observing the child, following the child’s lead, interpreting the child’s behaviour, parallel talk, and expanding and recasting the child’s language. The authors reported significant gains in parent communication behaviours and parental sensitivity post-intervention and noted that parents in the treatment group had children with significantly better language skills, when formally assessed three years post treatment.

A pilot RCT [34] involved parents receiving weekly, hour-long sessions for six months, where they were explicitly taught to use strategies to promote early communication. The authors referred to methods such as enhanced milieu teaching [101], prelinguistic milieu teaching [102], and The Hanen Program It Takes Two to Talk [100]. Examples of strategies include sitting face to face, using gestures, imitating/mirroring the child’s actions, and turn taking. The study reported that parents in the treatment group increased their use of communication support strategies by 17% compared to 2% in the control group., There was a large effect size of 1.09 (*p* = 0.03) for the difference in gains in deaf children’s prelinguistic speech skills between the treatment and control groups.

## 4. Discussion

This is the first systematic review focusing on research on the assessment of communicative parent behaviours within the context of parent–deaf child interaction. PCI is positively associated with improved child language in many at-risk hearing populations and in particular, within deafness [15]. The quality and quantity of interaction is an important predictor of a deaf child’s future language abilities [22,103]. However, it is not clear how to administer a good clinical assessment of PCI in deafness. Most of the included papers assess one or two aspects of PCI in isolation. This review condenses 40 years of research to provide us with details on the full range of parent behaviours that are assessed across the field of PCI in deafness and whether these behaviours correlate to higher levels of language in deaf children. We included a range of children’s hearing levels, a range of children’s communication mode, as well as hearing and deaf parents to capture a wider view of the interaction behaviours assessed between parents and their deaf children aged 0–3. This enabled us to avoid binary perspectives on parental interaction that is solely focused on oral-only or visual-only input, but instead we report on a combination of these alongside other important features such as joint engagement, emotional availability, warmth, and touch. In addition, this is also the first review of its kind to specifically detail the methods used in the assessment of PCI, with a view to develop the content of a future clinical assessment tool for PCI in deafness.

### 4.1. RQ1: What Parent Behaviours Are Being Assessed in Parent–Deaf Child Interaction?

Investigations have looked at a wide range of parent behaviours, including how a parent gains a child’s attention; the maintenance of attention in engagement; the emotional availability and responsivity of a parent during the interaction; and their strategies in providing accessible and stimulating linguistic input. These behaviours were purposely presented in the order they appear, with gaining a deaf child’s attention an important initial basis for subsequent successful interaction.

The four main areas of PCI uncovered in our review have some parallels with the review on children with language difficulties by Roberts and Kaiser [30], where the three most measured parent strategies were: parent responsiveness, use of language models and rate of communication. Similarly, a review by Holzinger and colleagues [104] on children with cochlear implants uncovered family involvement and parental linguistic input as key themes in their results. Additionally, within PCI research in the hearing population, the same set of behaviours are commonly measured [35].

Fourteen (14) of the papers (23%) identified in this review measured attention-getting behaviours and a further ten (16%) measured joint engagement. Joint attention skills predict receptive language [105] and are important to establish early. Whilst many papers in the hearing population assess how much a parent *re-directs* a child’s attention (as part of their parental directiveness) and how much time is spent in joint engagement, few researchers assess how a parent *gains* a child’s attention, except in the field of Autism [106]. It appears that the reduction or absence of hearing in deaf infants means the gaining of attention is a more prominent feature within PCI. This is presumably to increase access to parental signed or spoken language.

Within each theme highlighted in the review, there was much overlap between the parent behaviours identified across papers, despite differences in terminology. For example, ‘joint engagement’ and ‘joint attention’ were often used interchangeably in papers. We provided clear definitions of each parent behaviour within this review to facilitate collaborative working and a shared language between parents, Teachers of the Deaf, Speech and Language Therapists and academic researchers.

### 4.2. RQ2: How Are These Parent Behaviours Being Assessed?

The most prominent way of assessing PCI was with coding systems to analyse interactions. However, coding methods differed depending on the authors’ research focus. Some of the coding systems referred to well-known frameworks such as those from Waxman and Spencer [44], where attention-getting behaviours are well described and the coding scheme from Adamson, Bakeman, and Deckner [93], which includes 11 states of joint engagement. Other coding systems were created for the purposes of the particular study and papers did not report on the piloting of coding prior to their use.

Behavioural observation is the ideal method for assessing the quality of interactions and reduces the risk of bias that may arise from the use of self-reporting tools [107]. Lotzin et al. [35] also limited their review of PCI assessments to objective instruments, with all 24 of their included measures being validated rating scales. After coding, scales were the next and only other objective measure included within our review but only 11 papers (18%) used a validated scale.

### 4.3. RQ3: Which Parent Behaviours Are Correlated with Improved Child Language Outcomes?

Longer periods of joint engagement, increased parental sensitivity and a range of facilitative language techniques were all correlated with higher levels of language in deaf children. Parents with higher rates of maternal sensitivity and language stimulation have a greater effect on their child’s expressive language scores over time [13,64]. This finding is echoed in a systematic review by Holzinger et al. [104] where a meta-analysis of four longitudinal studies found that parental linguistic input explained 31.7% of the variance in deaf children’s expressive language scores. Their review included papers that also analysed parents’ verbal communication, whereas our review would have excluded these single modality studies. The findings in our review mirror not only those in deafness, but in the wider literature within the hearing population which reports that the quality and the quantity of parental talk is related to growth in children’s language [108].

Though attention-getting behaviours were assessed frequently within free-play PCI, no formal measure of child communication was administered within the 14 studies. We were therefore unable to uncover any relationships between formal language outcomes and getting a child’s attention. An exception is Tasker et al. [52], who monitored the success rates of maternal initiations for attention (i.e., how many bids resulted in the establishment of joint engagement) and found similar success amongst all three groups in their study (deaf children of hearing parents with CI, without CI, and hearing children of hearing parents). They did not include and compare deaf parents of deaf children.

An important correlation highlighted by Vohr et al. [66] was that parents with more support and higher SES had decreased intrusiveness, directiveness and negative regard. The better supported a parent is, the more sensitive, responsive, and positive they will be in their interactions. Hintermair [109] mirrored this finding in his study with parents of deaf children showing that child development profits from parents accessing ‘personal and social resources’ that influence their coping process and significantly lower stress. Furthermore, Zaidman-Zait et al. [110] found that higher levels of child acceptance were associated with lower levels of parenting stress in parents of deaf children.

### 4.4. Limitations of the Review Process

As described, we excluded papers that only analysed parents’ verbal interactions, that were not published in peer-reviewed journals, and were written in languages other than English. This may have led to some parent behaviours and/or methods of assessment being overlooked. However, when we compared our findings with those of the Holzinger et al. [104] systematic review, which used a different approach for their inclusion criteria, there were similar findings between the two papers.

For consistency and in order to compare data between studies, we only selected studies of PCI in the context of free-play. However, Mahoney, Spiker and Boyce [111] recommend that observations of PCI take place in a range of interactive contexts. It is also advised that interactions are assessed by an observer known to the dyad [112] and so, with many of our dyads being assessed cross-sectionally in play, by an unknown researcher, we must remain cautious when interpreting their findings.

The development of this research field and subsequent support and policy is driven by countries with more resources. In addition to being from countries of higher wealth, all the papers included in this review are from Western countries who share similar views on language acquisition. Further research on early language experiences in deafness and early language acquisition in diverse communities and plurilingual contexts is needed.

Though not a limitation as such, the majority of papers had a between-groups, observational design, where PCI was assessed at the same time as the child’s language. In these correlational studies we cannot attribute PCI as causing change in child language development. In order to identify causation and the predictive factors of PCI that benefit deaf children’s language outcomes, more RCTs, that extend the work of Roberts [34], are required.

## 5. Recommendations

Following this systematic review, we make the following recommendations for future research on PCI and deafness:

1. Provide full details with regard to participant information, for both the child and their parents including level of deafness, amplification use, child communication profile and parent-to-child communication profile (see ‘Language Access Profiles’ from Hall [113]).
2. Report all methodological details of parent–child interaction assessment including who filmed the PCI, location of the assessment, instructions given to parents, length of the recording and length of film analysed.
3. Use validated scales to assess PCI. We refer readers to Lotzin et al. [35] for their comprehensive list of psychometrically tested measures, where Biringen’s Emotional Availability Scales [96] are listed. This was the most commonly used validated scale in this review. In addition, reliability statistics should be reported.
4. Use frame-by-frame coding as a detailed method of analysis. Coding schemes should be explained in detail and their development and pilot testing described. Reliability statistics should be reported.
5. Recruit more representative samples of families with diverse socio-economic status and ethnicity.
6. Recruit and/or include deaf children with additional needs for similar reasons. The proportion of deaf children with additional needs is 22% [114].
7. Carry out more RCTs to explore causation between parental interaction and deaf children’s language growth.

### Clinical Implications

We recommend the following for professionals working in deafness:

1. Though frame-by-frame coding and testing reliability may not be appropriate activities for busy practitioners, knowing that eight minutes of interaction may provide enough data to use in discussion with parents is helpful and can reduce the need to film families for longer than this.
2. Scale measures may be a time-effective and efficient way of clinically measuring PCI, providing the scales are evidence based, valid and reliable.
3. Assessments of PCI should address attention-getting behaviours, states of joint engagement, parental sensitivity, and language input.
4. Assessments of PCI will assist in the planning of appropriately set, family-centred targets for intervention.
5. Practitioners should support parental stress and access to social resources following findings reporting the association between low levels of stress and higher quality PCI.

## 6. Conclusions

Good-quality PCI is widely acknowledged to be significant for child language development in deaf and hearing children. The outcomes within this review indicate that good-quality interaction requires the parent to: wait for or gain the child’s attention; maintain a shared, mutual focus with their child; follow their child’s lead; provide contingent and attuned responses to the child’s interests and needs; and use multi-modal methods to interpret, enrich and expand their child’s communicative attempts. Several of these behaviours have been associated with child language development outcomes. Yet, there are no specific clinical assessments for professionals to use in PCI with parents of deaf children. In order to inform the content of such an assessment, we carried out a systematic review of the PCI literature. This identified 61 papers that looked at interactions between hearing and deaf parents and their deaf children. These papers indicate that, over the past 40 years, there has been extensive attempts to document PCI with deaf children aged 0–3 years. However, many omissions in methodological reporting were noted, with the majority of studies lacking sufficient participant characteristics, details on setting, and data (video-recoding) length. Evaluations of PCI were conducted largely through detailed coding methods and the use of software—methods that are not typically available in routine clinical practice. A minority of studies assessed PCI via scales, although few of these were validated. This review provides the basis for the future development of an assessment tool for professionals to use in clinical contexts. Such a tool will facilitate the identification of targets for intervention and the monitoring of progress in parental communicative skills to maximise language learning opportunities for deaf children.

## Figures and Tables

**Table 2:** Methods of assessing PCI between included papers.

Method of Assessing PCI	*n* Papers (%)
Coding	40 (66%)
An existing, validated scale	7 (12%)
A novel scale	4 (7%)
A mix of validated and novel scales	2 (3%)
Coding and a validated scale	4 (6%)
Coding and a novel scale	4 (6%)

## Appendix A. Search Strategy

All eight databases were searched like this:

deaf OR deaf* OR ‘hearing impair*’ OR ‘hearing loss’ OR ‘hard of hearing’ OR d/hh OR dhh OR ‘cochlear implant’ OR ‘hearing aids’ OR ‘hearing disorders’

**AND**

child OR child* OR infant* OR baby or babies OR preschool OR kindergarten OR nursery OR toddler

**AND**

parent OR parent* OR caregiver OR ‘care giver’ OR mother OR father

**AND**

‘involvement’ OR ‘interaction’ OR ‘engagement’ OR ‘parent communication’ OR ‘parent engagement’ OR ‘child-directed interaction’ OR ‘facilitative communication’ OR ‘parent interaction characteristics’ OR ‘sensitivity’ OR ‘responsiv*’ OR ‘linguistic input’ OR ‘language input’ OR ‘relationship’ OR ‘communication support strategies’ OR ‘dyad’ OR ‘availability’ OR ‘intersubjectiv*’ OR ‘attention’ OR ‘attend’

Then, all data bases were searched again using the strategy below, except Scopus (unable to perform this function):

deaf OR deaf* OR ‘hearing impair*’ OR ‘hearing loss’ OR ‘hard of hearing’ OR d/hh OR dhh OR ‘cochlear implant’ OR ‘hearing aids’ OR ‘hearing disorders’

**AND**

child OR child* OR infant* OR baby or babies OR preschool OR kindergarten OR nursery OR toddler

**AND**

parent N5 (involvement OR interaction OR engagement OR communication OR ‘child-directed interaction’ OR ‘facilitative communication’ OR sensitivity OR responsiv* OR ‘linguistic input’ OR ‘language input’ OR ‘relationship’ OR ‘support strategies’ OR ‘dyad’ OR ‘availability’ OR ‘intersubjectiv*’ OR ‘attention’ OR ‘attend’

## Appendix B. Data Extraction Variables

**General**

Title

Publication year

Country of study

Conflicts of interest

Aims/Objectives

Study design

**Participants**

Total sample size—parents (*n*)

Sample size of hearing parents of deaf children

Sample size of hearing parents of hearing children

Sample size of deaf parents of deaf children

Sample size of deaf parents of hearing children

Total sample size (children)

Sample size of d/hh children

Sample size of hearing children

No. of dyads assessed

Participating groups (only CI, only HA, mix of HA/CI, mix of deaf/and NH)

Inclusion criteria

Exclusion criteria

**The child**

Age ranges of children in months

% of males

Unilateral or bilateral

Level of deafness (mild, moderate, severe, profound, not reported)

When deafness identified (months)

Aetiology of hearing loss

Amplification in use

Amplification provided (at age)

Additional needs (included, not included, unclear if children are included)

Language(s) used by child

Child exposed to sign lang?

**The adult**

Age of adult

Adult hearing status

Adult relationship to child

% females

Ethnicity (majority group, minority group, mixed group, other (state ethnicity), not stated, unclear)

Adult education level

Socio-economic status (mixed group, low SES, middle SES, high SES, not stated, unclear)

Language(s) used by adult

Language(s) used by adult to the child

Adult’s prev. experience of deafness

**The methods and procedure of the study:**

Multiple measured used? Y/N

Child measures/outcomes (i.e., communication, cognition, behaviour)

- Validated measures?
- Novel measures/devised for study?

Parent measures/outcomes (i.e., parental stress, self-efficacy)

- Validated measures?
- Novel measures/devised for study?

Measure used to assess parent–child interaction

- Coding
- Validated scale
- Novel measure/devised for study
- Other

If a tool/scale, number of items in the measure.

If a tool/scale, list the items in the measure.

Definitions of skills/constructs

Video—singular or a series (i.e., ‘one-off’ video or a collection of videos over time points?)

Software used for coding/analysis (i.e., ELAN, CLAN, INTERACT)

Length of interaction

Length of analysed section

Context of interaction (home, clinic, lab)

Coding method explicitly shared?

Reliability procedures

Blinding procedures

**If intervention study:**

Named intervention

Delivery of intervention (group, 1:1, modelling, coaching, other?)

Dose of intervention (no. of sessions, no. of weeks, length of individual session)

‘Control’ or alternative intervention?

**Results:**

Stats analysis used

Results on parent outcomes (T1/T2, means, median, range, scaled score, pre/post, change data)

Results on child outcomes (T1/T2, means, median, range, scaled score, pre/post, change data)

Results on PCI outcomes

**Discussion**

General summary

Confounding factors (identified by authors)

Limitations (identified by authors)

This paper was about (select all that apply: Touch, Sensitivity, Eye gaze, Emotional availability, Pointing, Linguistic input, Other—please type).

## Appendix C. List of Papers (*n* = 43) Excluded Based on Only Analysing Parent’s Verbal Interactions

Allen, S., Crawford, P., & Mulla, I. (2017). Exploring the acceptability of innovative technology: A pilot study using LENA with parents of young deaf children in the UK. *Child Language Teaching & Therapy*, *33*(2), 117–128, doi:10.1177/0265659016671168

Ambrose, S. E., Vandam, M., & Moeller, M. P. (2014). Linguistic input, electronic media, and communication outcomes of toddlers with hearing loss. *Ear & Hearing (01960202)*, *35*(2), 139–147, doi:10.1097/AUD.0b013e3182a76768

Benítez-Barrera, C. R., Angley, G. P., & Tharpe, A. M. (2018). Remote microphone system use at home: Impact on caregiver talk. *Journal of Speech*, *Language & Hearing Research*, *61*(2), 399–409, doi:10.1044/2017_JSLHR-H-17-0168

Benítez-Barrera, C. R., Thompson, E. C., Angley, G. P., Woynaroski, T., & Tharpe, A. M. (2019). Remote microphone system use at home: Impact on child-directed speech. *Journal of Speech*, *Language & Hearing Research*, *62*(6), 2002–2008, doi:10.1044/2019_JSLHR-H-18-0325

Bergeson, T. R. (2011). Maternal speech to hearing-impaired infants in the first year of hearing aid or cochlear implant use: A preliminary report. *Cochlear Implants International*, *12*(Suppl 1), S101-S104, doi:10.1179/146701011X13001035752741

Carey-Sargeant, C. L., & Brown, P. M. (2003). Pausing during interactions between deaf toddlers and their hearing mothers. *Deafness & Education International*, *5*(1), 39–58.

Carey-Sargeant, C. L., & Brown, P. M. (2005). Reciprocal utterances during interactions between deaf toddlers and their hearing mothers. *Deafness & Education International*, *7*(2), 77–97, doi:10.1179/146431505790560437

Caskey, M., & Vohr, B. (2013). Assessing language and language environment of high-risk infants and children: a new approach. *Acta Paediatr*, *102*(5), 451–461, doi:10.1111/apa.12195

Cheskin, A. (1981). The verbal environment provided by hearing mothers for their young deaf children. *Journal of Communication Disorders*, *14*(6), 485–496.

Costa, E. A., Day, L., Caverly, C., Mellon, N., Ouellette, M., & Ottley, S. W. (2019). Parent-child interaction therapy as a behavior and spoken language intervention for young children with hearing loss. *Language*, *Speech & Hearing Services in Schools*, *50*(1), 34–52, doi:10.1044/2018_LSHSS-18-0054

Cruz, I., Quittner, A. L., Marker, C., & Desjardin, J. L. (2013). Identification of effective strategies to promote language in deaf children with cochlear implants. *Child Development*, *84*(2), 543–559, doi:10.1111/j.1467-8624.2012.01863.x

DesJardin, J. L., & Eisenberg, L. S. (2007). Maternal contributions: supporting language development in young children with cochlear implants. *Ear & Hearing (01960202)*, *28*(4), 456–469.

Dirks, E., Stevens, A., Kok, S., Frijns, J., & Rieffe, C. (2020). Talk with me! Parental linguistic input to toddlers with moderate hearing loss. *Journal of Child Language*, *47*(1), 186–204, doi:10.1017/S0305000919000667

Fagan, M. K., Bergeson, T. R., & Morris, K. J. (2014). Synchrony, complexity and directiveness in mothers’ interactions with infants pre- and post-cochlear implantation. *Infant Behavior & Development*, *37*(3), 249–257, doi:10.1016/j.infbeh.2014.04.001

Ganek, H., Nixon, S., Smyth, R., & Eriks-Brophy, A. (2019). A Cross-cultural mixed methods investigation of language socialization practices. *Journal of Deaf Studies & Deaf Education*, *24*(2), 128–141, doi:10.1093/deafed/eny037

Ganek, H., Smyth, R., Nixon, S., & Eriks-Brophy, A. (2018). Using the Language ENvironment Analysis (LENA) system to investigate cultural differences in conversational turn count. *Journal of Speech*, *Language & Hearing Research*, *61*(9), 2246-2258, doi:10.1044/2018_JSLHR-L-17-0370

Harris, M. (2001). It’s all a matter of timing: sign visibility and sign reference in deaf and hearing mothers of 18-month-old children. *Journal of Deaf Studies & Deaf Education*, *6*(3), 177–185, doi:10.1093/deafed/6.3.177

Koester, L. S., Brooks, L. R., & Karkowski, A. M. (1998). A comparison of the vocal patterns of deaf and hearing mother-infant dyads during face-to-face interactions. *Journal of Deaf Studies & Deaf Education*, *3*(4), 290–301, doi:10.1093/oxfordjournals.deafed.a014357

Kondaurova, M. V., & Bergeson, T. R. (2011). The effects of age and infant hearing status on maternal use of prosodic cues for clause boundaries in speech. *J Speech Lang Hear Res*, *54*(3), 740–754, doi:10.1044/1092-4388(2010/09-0225)

Kondaurova, M. V., Bergeson, T. R., Huiping, X., & Kitamura, C. (2015). Affective properties of mothers’ speech to infants with hearing impairment and cochlear implants. *Journal of Speech*, *Language & Hearing Research*, *58*(3), 590–600, doi:10.1044/2015_JSLHR-S-14-0095

Kondaurova, M. V., Bergeson, T. R., & Xu, H. (2013). Age-related changes in prosodic features of maternal speech to prelingually deaf infants with cochlear implants. *Infancy*, *18*(5), 825–848, doi:10.1111/infa.12010

Kondaurova, M. V., Smith, N. A., Zheng, Q., Reed, J., & Fagan, M. K. (2020). Vocal turn-taking between mothers and their children with cochlear implants. *Ear & Hearing (01960202)*, *41*(2), 362–373, doi:10.1097/AUD.0000000000000769

Kristensen, N. M., Sundby, C. F., Hauge, M. N., & Lofkvist, U. (2020). Female caregivers talk more to 18-56-months-old children with and without hearing impairment than male caregivers measured with LENA (TM): A cross-sectional pilot study. *International Journal of Pediatric Otorhinolaryngology*, *130*, 9, doi:10.1016/j.ijporl.2019.109809

Lund, E. (2018). The effects of parent training on vocabulary scores of young children with hearing loss. *American Journal of Speech-Language Pathology*, *27*(2), 765–777, doi:10.1044/2018_AJSLP-16-0239

Majorano, M., Guidotti, L., Guerzoni, L., Murri, A., Morelli, M., Cuda, D., & Lavelli, M. (2018). Spontaneous language production of Italian children with cochlear implants and their mothers in two interactive contexts. *International Journal of Language & Communication Disorders*, *53*(1), 70–84, doi:10.1111/1460-6984.12327

Nicholas, J. G., & Geers, A. E. (2003). Hearing status, language modality, and young children’s communicative and linguistic behavior. *Journal of Deaf Studies & Deaf Education*, *8*(4), 422–437, doi:10.1093/deafed/eng029

Nienhuys, T. G., Cross, T. G., & Horsborough, K. M. (1984). Child variables influencing maternal speech style: Deaf and hearing children. *Journal of Communication Disorders*, *17*(3), 189–207, doi:10.1016/0021-9924(84)90011-X

Nienhuys, T. G., Horsborough, K. M., & Cross, T. G. (1985). A dialogic analysis of interaction between mothers and their deaf or hearing preschoolers. *Applied Psycholinguistics*, *6*(2), 121–139, doi:10.1017/S014271640000607X

Nittrouer, S., Lowenstein, J. H., & Antonelli, J. (2019). Parental language input to children with hearing loss: Does It matter in the end? *Journal of speech*, *language*, *and hearing research : JSLHR*, *63*(1), 234–258, doi:10.1044/2019_JSLHR-19-00123

Paradis, J., Blom, E., Szagun, G., & Schramm, S. A. (2016). Sources of variability in language development of children with cochlear implants: age at implantation, parental language, and early features of children’s language construction. *Journal of Child Language*, *43*(3), 505–536, doi:10.1017/S0305000915000641

Power, D. J., Wood, D. J., Wood, H. A., & MacDougall, J. (1990). Maternal control over conversations with hearing and deaf infants and young children. *First Language*, *10*(28, Pt 1), 19–35, doi:10.1177/014272379001002802

Roberts, M. Y., & Hampton, L. H. (2018). Exploring cascading effects of multimodal communication skills in infants with hearing loss. *Journal of Deaf Studies & Deaf Education*, *23*(1), 95–105, doi:10.1093/deafed/enx041

Saetre-Turner, M., Williams, C., & Quail, M. (2015). Caregiver-child interaction in children who are deaf or hard of hearing and children who are normally hearing: Preliminary data. *Journal of Clinical Practice in Speech-Language Pathology*, *17*(3), 144–150.

Smith, N. A., & McMurray, B. (2018). Temporal responsiveness in mother–child dialogue: A longitudinal analysis of children with normal hearing and hearing loss. *Infancy*, *23*(3), 410–431, doi:10.1111/infa.12227

Su, P. L., & Roberts, M. Y. (2019). Quantity and quality of parental utterances and responses to children with hearing loss prior to cochlear implant. *Journal of Early Intervention*, *41*(4), 366–387, doi:10.1177/1053815119867286

Sultana, N., Wong, L. L. N., & Purdy, S. C. (2020). Dataset on the calculations of daily adult word and conversational turn counts, and use of styles of oral interaction in 2-5-year olds with hearing loss in New Zealand. *Data Brief*, *30*, 105372, doi:10.1016/j.dib.2020.105372

Suskind, D. L., Graf, E., Leffel, K. R., Hernandez, M. W., Suskind, E., Webber, R., Nevins, M. E. (2016). Project ASPIRE: Spoken language intervention curriculum for parents of low-socioeconomic status and their deaf and hard-of-hearing children. *Otology & Neurotology*, *37*(2), e110–e117, doi:10.1097/MAO.0000000000000931

Szagun, G. (1997). A longitudinal study of the acquisition of language by two German-speaking children with cochlear implants and of their mothers’ speech. *Int J Pediatr Otorhinolaryngol*, *42*(1), 55–71, doi:10.1016/s0165-5876(97)00121-3

Szagun, G., & Rüter, M. (2009). The influence of parents’ speech on the development of spoken language in German-speaking children with cochlear implants. *Revista de Logopedia*, *Foniatría y Audiología*, *29*(3), 165–173, doi:10.1016/S0214-4603(09)70025-7

Thompson, E. C., Benítez-Barrera, C. R., Angley, G. P., Woynaroski, T., & Tharpe, A. M. (2020). Remote microphone system use in the homes of children with hearing loss: Impact on caregiver communication and child vocalizations. *Journal of Speech*, *Language & Hearing Research*, *63*(2), 633–642, doi:10.1044/2019_JSLHR-19-00197

Vandam, M., Ambrose, S. E., & Moeller, M. P. (2012). Quantity of parental language in the home environments of hard-of-hearing 2-year-olds. *Journal of Deaf Studies & Deaf Education*, *17*(4), 402–420, doi:10.1093/deafed/ens025

Vanormelingen, L., De Maeyer, S., & Gillis, S. (2015). Interaction patterns of mothers of children with different degrees of hearing: Normally hearing children and congenitally hearing-impaired children with a cochlear implant. *International Journal of Pediatric Otorhinolaryngology*, *79*(4), 520–526, doi:10.1016/j.ijporl.2015.01.020

Wang, Y., Jung, J., Bergeson, T. R., & Houston, D. M. (2020). Lexical repetition properties of caregiver speech and language development in children with cochlear implants. *Journal of Speech*, *Language*, *and Hearing Research*, *63*(3), 872–884, doi:10.1044/2019_JSLHR-19-00227

## Appendix D. List of Validated Scales Used for the Assessment of PCI

**Name of Validated Scale**	**Authors**	**Skills Assessed**	**Papers**
Emotional Availability Scales (4th ed.) (2008) [96]	Biringen, Z.	Parental Sensitivity	Used in 7/11 papers: Dirks et al. (2018); Lam-Cassettari et al. (2015); Pressman et al. (1998); Pressman et al. (1999); James et al. (2013); Paradis et al. (2015) and Pipp-Siegel et al. (1998).
Early Childcare Study Codes (Maternal Sensitivity and Cognitive Stimulation) (2000) [115]	National Institute of Child Health and Human Development Early Child Care Research Network.	Parental Sensitivity	Used in 2/11 papers: Quittner et al. (2013; 2016)
Parent Caregiver Involvement Scale (1986) [116]	Farran et al.	Parental Sensitivity	Used in 1/11 papers: Vohr et al., 2010
Communication-Promoting Behaviors Checklist for Caregivers (1992) [117]	Cole, E.	Parental Sensitivity and Parental Communication	Used in 1/11 papers: Nicastri, M. et al. (2020)
